# Research on a Service Touchpoint Design Model Driven by Smart Technology Based on Kano–Failure Modes and Effects Analysis

**DOI:** 10.3390/s24237854

**Published:** 2024-12-09

**Authors:** Jian Chen, Zhihan Li, Weiwei Wang, Yi Wang, Zhaoxuan He

**Affiliations:** School of Design and Art, Shaanxi University of Science & Technology, Xi’an 710026, China; ureycj@163.com (J.C.); 15591777672@163.com (Z.L.); wangyisj@sust.edu.cn (Y.W.); 18719883063@163.com (Z.H.)

**Keywords:** industrial design, service touchpoint, intelligent product development, failure analysis, intelligent product design decision

## Abstract

With the development of smart technology and the increasing variety of everyday products, factors influencing product service touchpoint design have become more diverse and complex. Existing service touchpoint design methods and models often focus narrowly on user research, co-design, and risk analyses, lacking a systematic approach. Consequently, they struggle to deliver solutions that align with user needs. This misalignment may result in issues such as increased cognitive load during product use, a diminished user experience, and lower evaluations of the product. In response, this paper proposes a service touchpoint design model, the “BEDFITA” model. It starts with user behavior and follows a structured, systematic process that includes understanding user behavior, recording user emotions, matching user needs, designing product functions, planning interaction experiences, designing service touchpoints, and analyzing failure risks. The Kano model is employed in the user requirement identification phase to provide more precise user requirement parameters, while FMEA is employed in the failure risk analysis phase to generate more accurate failure risk assessments. This ensures that the final service touchpoint design meets user needs and offers reliability and robustness. Finally, the feasibility and effectiveness of the proposed model are validated through a case study on the service touchpoint design of a smart desk.

## 1. Introduction

The essence of service touchpoint design is to shape the complete user behavior during activities such as daily life, work, entertainment, leisure, healthcare, or learning. This behavior encompasses several stages: the intention phase before the activity, the planning phase, the action phase, the completion phase, and finally, the evaluation phase. Service touchpoints are the tangible carriers through which products provide users with visual, tactile, auditory, olfactory, gustatory, emotional, and imaginative experiences at each stage [1]. The form, shape, color, material, and other characteristics of these tangible carriers are significantly influenced by user preferences and needs within their complete behavior pattern. As a result, due to the diverse preferences and behaviors of different users, the design of service touchpoints is inherently varied and complex [2].

With the development of smart technology, several service touchpoints have acquired intelligent attributes. Compared to traditional touchpoints, those with smart features offer users more varied interaction behaviors and modes, resulting in a more convenient, comfortable, and seamless user experience [3]. While smart technology has the potential to enhance user experience, it also increases the complexity of service touchpoint design. This growing complexity necessitates a systematic design model when designing smart touchpoints [4]. Such a model must take user behavior as the starting point, extracting user needs based on the emotions experienced at each behavioral stage. The product’s functions and tangible carriers should be designed according to these needs. Finally, this process ensures a service touchpoint design that meets user requirements, resulting in a more convenient, intuitive, efficient, and comfortable user behavior, thereby improving the overall user experience.

The current design of service touchpoints largely relies on the experience of designers and traditional product design methods. The design process typically focuses on aspects such as the shape, size adjustment, and material selection of service touchpoints, but it lacks a foundation in research on user behavior and user needs to inform the design of touchpoint interactions. As a result, many existing products fail to meet user needs or are not aligned with user behavior habits, leading to confusion and dissatisfaction during use. In some cases, designs that do not align with user behavior habits may even mislead users, causing operational errors and unnecessary negative impacts. Therefore, research on service touchpoint design methods and models driven by smart technology is both necessary and significant.

## 2. Research Status

Current research on user needs and user behavior primarily revolves around the Kano model and the FMEA (Failure Modes and Effects Analysis) method. Design researchers typically use the Kano model to capture user requirements, while the FMEA method is employed to analyze potential failure modes and risks in both the design and manufacturing processes, as well as during product use.

The Kano model is an efficient tool for classifying and quantifying user expectations, offering a more precise identification of user needs. It has been widely used in product design for user needs assessments, emotional analyses, and design critiques, as well as in industries such as healthcare, management, and services for analyzing personnel needs. For instance, Yao et al. [5] used the Kano model to design a questionnaire for hospital emergency departments, which successfully captures patient needs and improves the patient experience. To verify the feasibility of this method, a sample of 100 people was employed at Guizhou Provincial People’s Hospital. Using the Kano model, 19 patient needs were identified and categorized, effectively improving the patient experience. Similarly, Zhang et al. [6] applied the enhanced Kano model to address customer needs in the e-commerce field. By extracting customers’ expectations from product reviews, they further identified user needs, aiding subsequent product updates and iterations. Moreover, Xiao et al. [7] designed a survey questionnaire using the Kano model to address social demands for aging health management amidst accelerating global aging. They collected data from 143 users and analyzed it across four dimensions, ultimately determining the design direction for aging health management platforms and offering development and optimization strategies for such platforms. Adding to that, Ce et al. [8] conducted a survey of local supermarket consumers using the Kano–QFD model to increase competition in the retail market. This approach aligned supermarket service offerings with consumer needs and optimized the retail service experience to meet the rising demands for service content and quality. In addition, Wen et al. [9] employed the Kano model as a framework to study tourists’ preferences and emotional needs for creative products at a cultural memorial hall. They designed a series of cultural creative products to enhance the hall’s cultural influence and publicity. Furthermore, Qi et al. [10] discussed users’ emotional needs for health water bottles, emphasizing the strong correlation between user senses and product design. By combining the Kano model with the AHP model, they piloted a quantitative analysis of user needs, extracting design elements to serve as a basis for proposals and providing designers with an effective method for innovation. Moreover, Hariri et al. [11] improved the Kano model and the SWARA method to analyze 112 Customer Requirements (CRs), classifying them into different attributes. This improved production systems in the automotive and manufacturing industries, enhancing product quality and market competitiveness. Finally, Dai et al. [12] designed a Kano–AHP model to build an industrial product design and service platform. By analyzing and computing user needs and performance, they verified the platform’s effectiveness in meeting customer service demands.

The Failure Mode and Effects Analysis (FMEA) method, along with the Process Potential Failure Mode and Effects Analysis (PPFMEA) method, is a systematic, proactive tool used for risk management to prevent potential issues. In recent years, local and international scholars have applied FMEA to service design, offering guidance on improving service effectiveness. For instance, Haizhe et al. [13] addressed the limitations of FMEA in medical device design by proposing a structured human reliability assessment model, which optimizes a failure risk analysis by introducing scenario parameters. This improvement resolved issues of numerical repetition and weight allocation encountered in traditional FMEA, resulting in more accurate and reliable medical device design. Moreover, I. D. P et al. [14] enhanced the failure risk analysis in FMEA by aligning different detection methods with various types of risks and incorporating a comprehensive impact of different factors to determine failure probabilities. Adding to that, Padash et al. [15] introduced a hybrid fuzzy risk assessment method for the high-risk steel industry, which more accurately quantifies expert judgments in uncertain environments, refining FMEA’s risk assessment outcomes and effectively lowering risk incidents. Furthermore, Kornek et al. [16] highlighted the shortcomings of FMEA in radiotherapy risk assessment, and by testing and evaluating a new event reporting system (IRS), demonstrated its ability to validate FMEA results. In addition, Barsalou et al. [17] improved FMEA’s analytical structure by dividing the process into system analysis, failure analysis, risk mitigation, and risk communication stages. They refined each stage through a seven-step method, enhancing the systematic nature of FMEA. Moreover, Wencai et al. [18] discussed improved vehicle fault mode evaluation from the perspective of user driving experience. They noted that traditional FMEA methods struggle to achieve this task. Therefore, they combined cost FMEA, FAHP, and EFMULTIMOORA. Cost FMEA was used to identify vehicle fault modes, FAHP calculated the weights of various criteria, and EFMULTIMOORA classified these fault modes. Finally, Heiko et al. [19] discussed the importance of fault detection and risk ranking in Industry 4.0 and introduced several methods and models for fault detection and risk ranking, highlighting FMEA’s effectiveness in analyzing failure risks in product design and manufacturing.

The research outcomes mentioned above provide valuable insights into product service touchpoint design, smart-driven product design, and user experience optimization methods. Additionally, they offer several decision models for design research. However, in the context of smart technology-driven product design, there is a lack of systematic design models and processes. Existing methods are often too narrow in scope, making it difficult to address the evolving design challenges of intelligent products. Consequently, many market products fail to meet user expectations. To address this gap, the BEDFITA model is proposed for designing product service touchpoints in smart technology applications. This model organizes the design process into phases, systematically outlining research objectives, methods, and outcomes for each phase. This approach enhances the overall integrity, reliability, and feasibility of product design. Ultimately, solutions generated through the BEDFITA model better align with user needs, leading to an enhanced user experience.

## 3. Service Touchpoint Design Driven by Smart Technology

Smart technology, rooted in statistical methods, integrates disciplines such as big data, the internet, the Internet of Things (IoT), and neural networks to emulate human intelligence. This allows smart technology to endow artificial objects, such as products, tools, and software, with intelligent, human-like characteristics [20]. In the realm of product service touchpoint design, the advancement of smart technology has transformed the way humans interact with products. The relationship between users and products has evolved from a complex “control and be controlled” dynamic to one of “inquiry and learning”. Consequently, smart technology-driven product service touchpoint design provides a wider range of interaction options, while increasing the diversity and complexity of these touchpoints. Under these conditions, traditional design approaches, which rely primarily on designers’ experience, often struggle to ensure the reliability of design solutions. Single-stage design models and methods are insufficient to guarantee that the solutions meet user needs [21].

Users interact with product functions to solve problems in social and survival activities, which is the fundamental purpose of using a product. Similarly, these interactions are at the core of the user experience, with service touchpoints being the focal points of these interactions, heavily influenced by user behavior preferences. Therefore, product service touchpoint design must be user-centered, beginning with an analysis of user behavior and the emotions they experience while addressing issues in their daily lives. This approach uncovers user needs across different attributes, enabling the creation of design solutions that meet those needs. To this end, this paper proposes the BEDFITA model, a comprehensive framework for designing product service touchpoints. This model includes a user behavior analysis, emotion recording, needs matching, product function design, interaction experience planning, service touchpoint design, and a failure risk analysis. By adopting this structured approach, the design process becomes more holistic and logical, ensuring that design solutions better align with user needs to optimize the user experience.

Moreover, the BEDFITA model is divided into two phases:

(1) Function Design Phase Based on User Behavior: Start by clearly identifying the specific behaviors users exhibit when interacting with a product or engaging in an activity. This involves systematically mapping the complete behavioral process, from the starting point to the endpoint of the interaction. For each behavioral stage, employ the Kano model to ask both positive and negative questions, capturing users’ emotions and reactions at each stage. Based on users’ varying emotions at different behavioral points, identify and match their specific needs. This information is used to design the product’s functions.
(2) Service Touchpoint Design and Failure Analysis Phase Based on FMEA: Using the functional design results from the Function Design Phase, a comprehensive interaction experience map is created for users. Next, an appropriate service touchpoint is selected for each interaction behavior point in the interaction experience map, resulting in a complete mapping of the product’s service content and touchpoints. To ensure reliability, a failure risk analysis is conducted on each service touchpoint using FMEA. If any service touchpoint presents a high failure risk, re-evaluate and select alternative touchpoints until all pass the failure risk analysis stage. This process leads to a robust, failure-resistant service touchpoint design solution.

**Figure 1:**
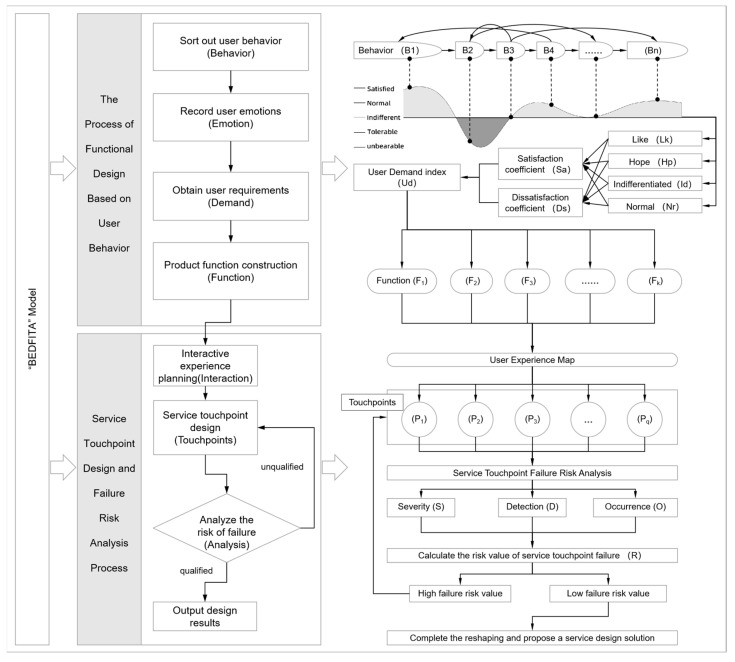
Technical route map of BEDFITA model.

The model is named the BEDFITA model, as it follows a sequential logic of key terms: Behaviors → Emotions → Demands → Functions → Interaction → Touchpoints → Analysis. The specific technical framework of this model is illustrated in Figure 1.

## 4. Process of Constructing Functional Solutions Based on User Behavior

User behavior is the starting point and core focus for constructing product function solutions. In this paper, user behavior mainly refers to two aspects: the interaction behavior when using a product and specific behaviors during survival and production activities [22]. When users interact with a product, due to factors such as design flaws in the product, new demands driven by market competition, and users’ evolving needs, the original product may no longer provide optimal service. This reduces the efficiency and experience of user interaction. For example, compared to feature phones with physical buttons, smartphones introduced a new interaction behavior for users, offering more efficient and improved user experiences than button-based phones [23].

In survival and production activities, the lack of assistance can result in lower behavioral efficiency and poor user experiences. For example, when children are learning to walk upright, they require support and protection, leading to the design of products like baby walkers [24].

User behavior generates emotions, and these emotions reflect user needs. Naturally comfortable user behavior generates relaxed and pleasant emotions. Under such emotional states, the user’s need may be to prolong the activity. Conversely, prolonged and complex user behavior results in fatigue, and under such emotional states, the user may require a reduction in the complexity of the behavior or seek to avoid the behavior altogether. At this point, the various functions of a product are needed to meet the user’s needs under different emotional states [25].

As described above, the process of constructing a product’s function solution must begin by organizing and analyzing user behavior, recording the emotions generated by these behaviors. The Kano model is then used to describe and quantify these emotions, revealing the various user needs reflected in different emotional states. Finally, product function solutions are developed based on these user needs.

**Figure 2:**
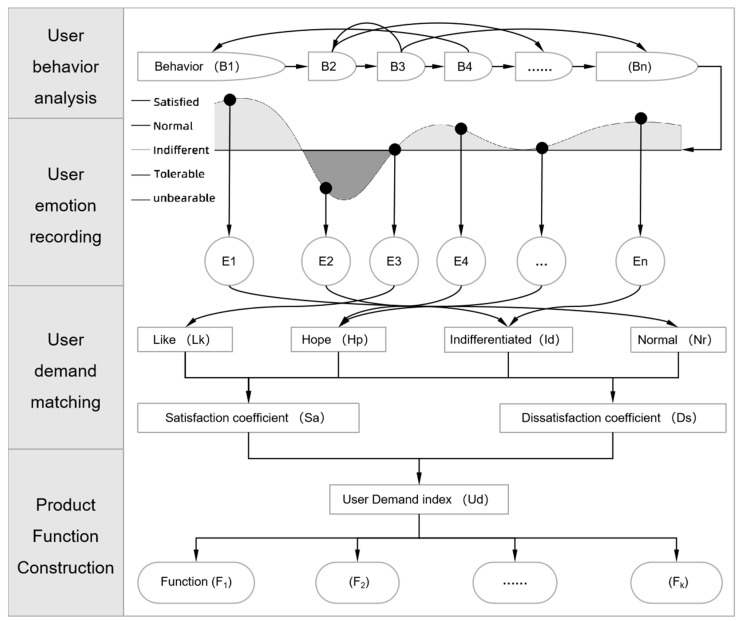
Functional solution construction process.

The process of constructing function solutions based on user behavior is illustrated in Figure 2.

The specific steps and content of constructing functional solutions are as follows:

### 4.1. User Behavior Analysis

To design product service touchpoints driven by smart technology, it is essential to thoroughly understand user behavior when using a specific product or engaging in a specific activity. This behavior comprises multiple behavioral nodes. Users typically begin at a fixed behavioral node, may traverse or skip through intermediate nodes, and ultimately conclude at another fixed behavioral node. Therefore, the goal of analyzing this complete behavior is to ensure the sequence of each behavioral node.

Let user behavior be denoted as B, and the m-th (where m = 1, 2, 3, …, n) behavioral node is denoted as B_m_.

### 4.2. User Emotion Recording

Throughout the process of engaging in the aforementioned behavior, users will experience different emotions at each behavioral node. Each node has a corresponding relationship with the user’s emotions. Therefore, user emotions are denoted as E, and the emotion experienced by the user at the m-th behavioral node is denoted as E_m_.

### 4.3. User Demand Matching

Based on the Kano model, user emotions at each behavioral node are categorized into the following: Like (Lk), Neutral (Nr), Expectation (Hp), Indifference (Id), Confusion (Ss), and Dislike (Ng). Each behavioral node has two dimensions: “With this behavior” and “Without this behavior”. User evaluations for each dimension can be classified as “Satisfied”, “Normal”, “Indifferent”, “Tolerable”, and “Dissatisfied”. A user demand matching table was created to record the emotions associated with each behavioral node, serving as the basis for aligning user demands. This table is shown in Table 1.

Let the user group participating in this user demand matching record be denoted as H, with the number of users denoted as h, where i = 1, 2, 3, …, h. Let Eim represent the evaluation of the i-th user for the m-th behavioral node. Users’ evaluations for each behavioral node are recorded sequentially in the user demand matching table. After completing this table, the ratio of each type of user emotion to the number of users for each behavioral node is computed and expressed as a percentile, as shown in Equation (1).

(1) $${Lk}_{m}\%+{Hp}_{m}\%+{Nr}_{m}\%+{Id}_{m}\%+{Ng}_{m}\%+{Ss}_{m}\%=100\%$$

where represents the percentage of the user group who expressed “Like” for this behavioral node, denotes the percentage of users who expressed “Expectation”, indicates the percentage of users who expressed “Neutral”, represents the percentage of users who expressed “Indifference”, is the percentage of users who expressed “Dislike”, and highlights the percentage of users who expressed “Confusion” for this behavioral node.

The satisfaction coefficient Sam is introduced to represent users’ satisfaction when the m-th behavior is present, and the dissatisfaction coefficient Dsm is introduced to represent user dissatisfaction when the m-th behavior is absent. The user demand coefficient at the m-th behavioral node is denoted as Udm, and the specific calculations are shown in Equations (2)–(4) [26].

(2) $${Sa}_{m}=\frac{{Hp}_{m}\%+{Lk}_{m}\%}{{Hp}_{m}\%+{Lk}_{m}\%+{Id}_{m}\%+{Nr}_{m}\%}$$

(3) $${Ds}_{m}=\frac{{Nr}_{m}\%+{Lk}_{m}\%}{{Hp}_{m}\%+{Lk}_{m}\%+{Id}_{m}\%+{Nr}_{m}\%}$$

(4) $${Ud}_{m}={Sa}_{m}+{Ds}_{m}$$

The design of product functions must closely align with user needs. Functions based solely on designers’ experience may lead to wasted functionality and increased costs. From a user experience perspective, features that do not address user needs can adversely affect users’ judgments and behaviors. Therefore, the demand coefficients obtained above should be ranked, and product functions should be developed according to this ranking to create an effective product function plan. Product functions are denoted as F, with the j-th product function represented as F_j_ (where j = 1, 2, 3, …, k). The product function solution constructed at this stage will serve as the foundation for the subsequent service touchpoint design.

## 5. Service Touchpoint Design and Failure Risk Analysis Process

In the previous discussion, we completed the construction of the product function solution based on user behavior, resulting in a solution that contains k different functions. However, when users interact with a product’s functions, they can choose from various interaction methods. For example, controlling water flow is a function of a faucet, and within this function, there are multiple interaction options such as turning a knob, toggling a valve, or using smart sensors [27]. Due to the variation in interaction methods, the specific form of the product’s service touchpoints will also differ. For instance, the interaction method of turning a knob requires the service touchpoint to be in the form of a knob, whereas the smart sensor interaction method necessitates a motion-capture camera as the service touchpoint form [28]. Therefore, the design of service touchpoints must be based on the specific product function solution. First, based on the function solution, we sequentially plan the different interaction methods users will employ when using all functions, resulting in a complete interaction process for the user. Then, for each interaction method corresponding to the product functions, we sequentially design the form of the service touchpoints, leading to the creation of a service touchpoint design plan [29].

During the interaction between the user and the product’s service touchpoints, the touchpoints remain subject to failure risks. A failure in the service touchpoint could impair certain product functions and disrupt the interaction between the user and the product, reducing the overall user experience. Therefore, once the service touchpoint design plan is completed, it cannot proceed directly to production and manufacturing without conducting a failure risk analysis. Due to factors such as the user’s knowledge, the form of the service touchpoint, and other variables, the failure risks of service touchpoints are uncertain. To address this, the FMEA (Failure Modes and Effects Analysis) method is applied to detect the failure causes of each touchpoint in the design plan, analyze its failure modes, and predict the potential consequences of its failure.

The failure risks of service touchpoints are quantified by evaluating the severity (S) of failure risks, the detectability (D) of failure causes, and the occurrence (O) frequency of failure modes [30]. This process yields a failure risk value for each touchpoint. Touchpoints with high failure risk values have lower reliability. For such touchpoints, they are returned to the corresponding design phase for redesign. The new design plan undergoes a failure risk analysis, and this iterative process is repeated until the failure risk value decreases. Alternatively, reliability can be ensured through a design that improves robustness, allowing normal interaction between the user and the touchpoint. Touchpoints with low failure risk values exhibit high reliability, so their design plans are retained. Following this process, the final output is the product’s service touchpoint design result [31].

**Figure 3:**
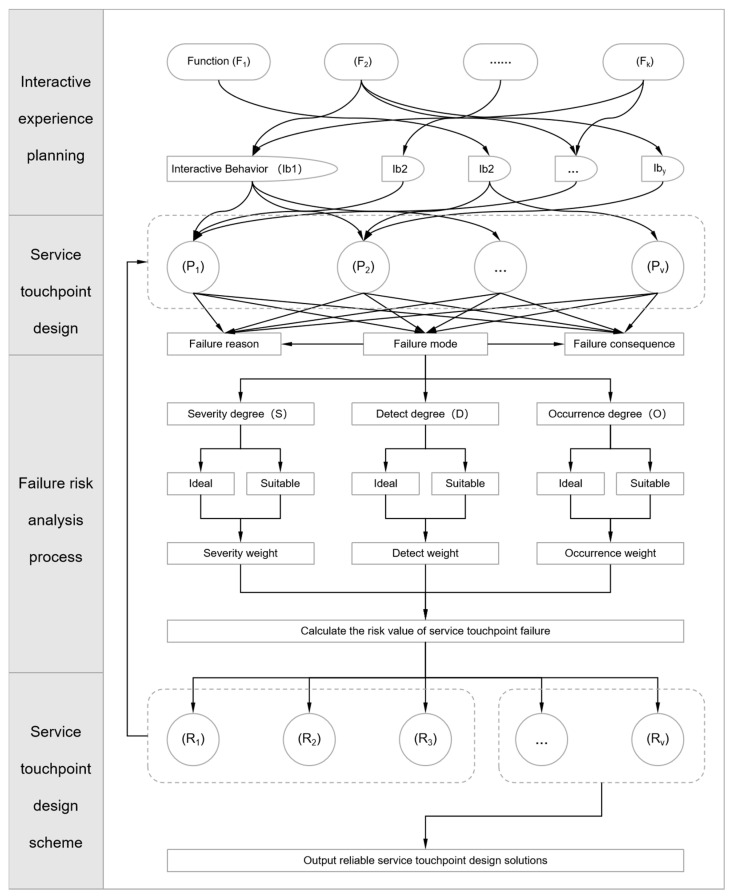
Service touchpoint design and failure risk analysis process.

The flow of the service touchpoint design and failure analysis is illustrated in Figure 3.

### 5.1. Interaction Behavior Planning

The design of product service touchpoints should first plan user interaction behaviors for each product function according to the functional solution constructed earlier. The relationship between user interactions and product functions is not always one to one. Multiple interactions may correspond to a single product function. For example, when using a smartphone, various interactions such as video calls, watching movies, and browsing information all correspond to the phone’s image display function. At the same time, a single interaction can correspond to multiple product functions. For instance, when using a smart desk lamp, the single action of repeatedly touching the light adjustment button can correspond to several functions, including adjusting the color temperature, brightness, lighting effects, and setting a timer [32].

Therefore, based on the k product functions included in the functional solution, user interaction behaviors should be planned to ensure that each interaction for using the product functions is as easy to understand, easy to adapt to, and as healthy and comfortable as possible. The relationship between product functions and interaction behaviors is illustrated in Figure 3. The total interaction behavior for using all product functions is denoted as Ib, where Ib_z_ represents the z-th user interaction behavior (z = 1, 2, 3, …, y).

### 5.2. Service Touchpoint Design

The user experience journey map outlines the complete flow of user interaction with product functions to achieve their goals. This interaction flow comprises multiple interaction behavior nodes, each requiring material mediums based on visual, tactile, auditory, olfactory, and gustatory inputs. These material mediums are known as service touchpoints. Based on the interaction experience flow developed earlier, service touchpoints should be selected or designed for each behavior node to create a detailed user interaction behavior journey map. This journey map illustrates the service touchpoints associated with each interaction behavior node, collectively forming a provisional service touchpoint design plan. Service touchpoints are denoted as P, with P_w_ representing the w-th service touchpoint (where w = 1, 2, 3, …, v). This provisional design plan will undergo a failure risk analysis in the subsequent steps.

### 5.3. Service Touchpoint Failure Risk Analysis

To assess the reliability and stability of the service touchpoint design plan, a failure analysis is required. The FMEA method is commonly employed to identify risks in service systems, product design, and user experience processes [33]. Therefore, this paper utilizes the FMEA method to analyze potential failures for each service touchpoint in the design plan.

Since user evaluations often contain ambiguous terms such as “should”, “seems”, “particularly”, “very”, and “slight”, this paper employs triangular fuzzy numbers for the calculation and analysis. A unified evaluation standard or fuzzy term set is constructed for the severity (S), occurrence (O), and detection (D) of failures in the FMEA method. These three indicators are treated as fuzzy linguistic variables. A fuzzy linguistic term set with five evaluation terms is used for each variable, with scores ranging from [0, 10]. The corresponding fuzzy evaluation terms are displayed in Table 2, where the minimum fuzzy number is L, the middle fuzzy number is N, and the maximum fuzzy number is U.

Triangular fuzzy numbers, introduced by Zadeh in 1965 as part of the fuzzy sets theory, address uncertainties in various environments. They are extensively used in quality and risk management due to their ability to accurately represent relationships between elements and sets within fuzzy sets [34]. Therefore, triangular fuzzy numbers are employed to quantify user evaluations in the failure analysis of service touchpoints. The membership function for the fuzzy terms listed in Table 2 is used to quantify and describe user evaluations. The membership function for a user evaluation X is given by Equation (5) as follows:

(5) $$\mu Ã(x)\left\{\begin{array}{c} \frac{x-L}{N-L}\;\;\;\;\;\;\;\;\;\;L\leq x\leq N\\ \frac{U-c}{U-N}\;\;\;\;\;\;\;\;\;\;N<x\leq U\\ 0\;\;\;\;\;\;\;\;\;\;\;\;\;\;\;\;\;\;\;\;Other \end{array}\right.$$

For the evaluation of the failure risk of a service touchpoint by the i-th user, denoted as C_i_, the triangular fuzzy number is expressed as follows:

(6) $$\left\{\begin{array}{c} L=\sum_{i=1}^{h}a_{i}L_{i}\\ N=\sum_{i=1}^{h}a_{i}N_{i}\\ U=\sum_{i=1}^{h}a_{i}U_{i} \end{array}\right.$$

where $\sum_{i=1}^{h}c_{i}=1,c_{i}\in (0,1)$.

To perform quantitative comparisons between the failure risks of different service touchpoints, defuzzification is a crucial step. Various methods are available for defuzzification, with the centroid method offering a relatively accurate output. The centroid method determines the final defuzzified value by finding the centroid of the area under the membership function curve and the horizontal axis. This centroid represents the defuzzified failure risk value of the service touchpoint. Therefore, the defuzzification calculation process is defined as follows:

(7) $$x(Ã)=\frac{\int_{L}^{U}x\mu Ã(x)dx}{\int_{L}^{U}\mu Ã(x)dx}$$

To calculate the fuzzy numbers for the failure risk factors in the FMEA of service touchpoints, a_Si_, a_Oi_, and a_Di_ represent the weight values of severity, occurrence, and detection, respectively, for the i-th user. Moreover, S_mi_, O_mi_, and D_mi_ denote the fuzzy numbers corresponding to the severity S, occurrence O, and detection D evaluation levels of the failure mode for the i-th user at the w-th service touchpoint. The fuzzy numbers for the severity, occurrence, and detection of the w-th service touchpoint are denoted by S_w_, O_w_, and D_w_, respectively, and are calculated using Equations (8)–(10):

(8) $$S_{w}=\sum_{i=1}^{h}a_{si}S_{wi}=(\sum_{i=1}^{h}a_{si}S_{wiL},\sum_{i=1}^{h}a_{si}S_{wiN},\sum_{i=1}^{h}a_{si}S_{wiU})$$

(9) $$O_{w}=\sum_{i=1}^{h}a_{Oi}O_{wi}=(\sum_{i=1}^{h}a_{Oi}O_{wiL},\sum_{i=1}^{h}a_{Oi}O_{wiN},\sum_{i=1}^{h}a_{Oi}O_{wiU})$$

(10) $$D_{w}=\sum_{i=1}^{h}a_{Di}D_{wi}=(\sum_{i=1}^{h}a_{Di}D_{wiL},\sum_{i=1}^{h}a_{Di}D_{wiN},\sum_{i=1}^{h}a_{Di}D_{wiU})$$

where S_wiL_, O_wiL_, and D_wiL_ denote the minimum fuzzy numbers for severity, occurrence, and detection, respectively, assigned by the i-th user for the w-th service touchpoint. Moreover, S_wiN_, O_wiN_, and D_wiN_ represent the intermediate fuzzy number for severity, occurrence, and detection, respectively, assigned by the i-th user for the w-th service touchpoint. Finally, S_wiU_, O_wiU_, and D_wiU_ present the maximum fuzzy numbers for severity, occurrence, and detection, respectively, assigned by the i-th user for the w-th service touchpoint.

### 5.4. Output of Service Touchpoint Design Plan

To quantitatively compare the failure risks of different service touchpoints, the evaluation results were deblurred using the centroid method, as described in Equation (7). The triangular fuzzy numbers for the severity, likelihood, and detectability of failure modes at service touchpoints, along with the deblurring process, are expressed in Equations (11), (12), and (13), respectively.

(11) $${QS}_{w}=\frac{1}{3}\left[\left(S_{wU}\right.-\left.S_{wL}\right)+\left(S_{wN}\right.-\left.S_{wL}\right)\right]+S_{wL}$$

(12) $${QO}_{w}=\frac{1}{3}\left[\left(O_{wU}\right.-\left.O_{wL}\right)+\left(O_{wN}\right.-\left.O_{wL}\right)\right]+O_{wL}$$

(13) $${QD}_{w}=\frac{1}{3}\left[\left(D_{wU}\right.-\left.D_{wL}\right)+\left(D_{wN}\right.-\left.D_{wL}\right)\right]+D_{wL}$$

where Q_Sw_, Q_Ow_, and Q_Dw_ represent the severity, occurrence, and detection measures of the failure risk of the w-th service touchpoint after processing with fuzzy theory. Moreover, S_wL_, O_wL_, and D_wL_ denote the minimum fuzzy numbers obtained when assessing the severity, occurrence, and detection levels of the failure mode of the w-th service touchpoint. In addition, S_wN_, O_wN_, and D_mN_ represent the intermediate fuzzy numbers obtained during the same assessment. Finally, S_wU_, O_wU_, and D_wU_ denote the maximum fuzzy numbers obtained during the assessment of the severity, occurrence, and detection levels of the failure mode of the w-th service touchpoint.

Based on the user’s evaluation of the failure risk assessment for service touchpoints, and after performing fuzzification and defuzzification, the failure risk value Rw for the w-th service touchpoint is determined. The calculation process is detailed in Equation (14).

(14) $$R_{w}={QS}_{w}\times {QO}_{w}\times {QD}_{w}$$

According to Equation (14), the failure risk values R_1_, R_2_, …, R_v_ are sequentially obtained for P_1_, P_2_, …, P_v_. Service touchpoints with lower failure risk values exhibit higher reliability and can be directly used as solutions. In contrast, service touchpoints with higher failure risk values demonstrate lower reliability and must be returned to the design phase for redesign. These redesigned touchpoints should then undergo a failure risk analysis again to verify their reliability and stability.

## 6. Case Study Verification

An office desk is a piece of equipment designed to facilitate collaborative work and social activities, making it convenient for office workers and their tasks. With advancements in smart technology, office desks have begun to incorporate intelligent features, offering users more efficient ways to work. In light of this, this case study examines the design process of service touchpoints for smart office desks, applying the BEDFITA model proposed in this paper [35].

### 6.1. Function Construction of Office Desk Driven by Smart Technology

Initially, we employ non-participant observation methods to investigate and analyze user behavior during office activities. This analysis results in a comprehensive overview of office behavior, comprising 19 behavioral nodes, as detailed in Table 3.

**Figure 4:**
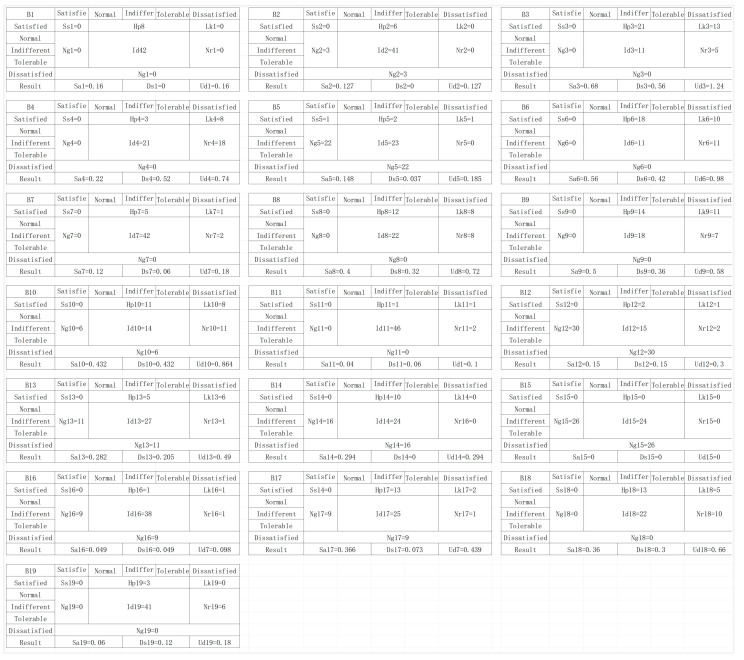
Calculation results of user demand coefficients for office behavior nodes.

The core user group H for office desks was selected, comprising 50 users. Through a questionnaire survey, user evaluation values were sequentially obtained based on the order of behaviors identified in the user office behavior analysis. Core users assessed each behavior node with a binary evaluation of “present/not present”, and rated their satisfaction using terms such as “Satisfied”, “As it should be”, “Indifferent”, “Tolerable”, and “Dissatisfied”. The results of these evaluations were used to calculate the demand coefficient for each user office behavior node, as described in Equations (1)–(4) previously mentioned. The data obtained are shown in Figure 4.

By sorting the demand coefficients of office behavior nodes from highest to lowest, the primary needs of users during office activities are identified. Based on these needs, the functions of the office desk are constructed, as shown in Table 4.

### 6.2. Service Touchpoint Design for Office Desks Driven by Smart Technology

After achieving the function construction of the office desk, and based on the smart office desk’s functional plan, the user’s experiential behaviors are mapped out when using the above functions and the corresponding service touchpoints are designed. The preliminary textual description of the office desk service touchpoint design plan is shown in Table 5.

A failure risk analysis is conducted for the aforementioned service touchpoints. Given the diverse professions within the core user group, their work contents vary, affecting the accuracy of user weight assignments. Therefore, the core user group is divided into four major professional groups. By accounting for the differences in professions and work content among these groups, we determine the user weight assignments for the failure severity, occurrence, and detection of the service touchpoints. Using Equations (5)–(7), we sequentially defuzzify the initial user weight assignments to generate the final user weights, as detailed in Table 6.

Through a collaborative analysis of core users, the potential failure modes for service touchpoints P1–P8 were identified. The potential causes of these failures were traced, and their impacts and consequences were predicted. A consensus was reached regarding the failure modes and potential risk causes for each service touchpoint, as displayed in Table 7.

Based on the scoring standards in Table 2, the fuzzy levels of the severity, occurrence, and detection of the failure modes for each service touchpoint are evaluated. Combining these findings with the expert weight matrix in Table 6, the severity, occurrence, and detection of the service touchpoints are analyzed using fuzzy logic. To generate more accurate results, the fuzzy numbers for the three parameters are calculated sequentially using Equations (8)–(10), and the results are illustrated in Table 8.

Using Equations (11)–(13), the fuzzy numbers for the severity, occurrence, and detection of the failure modes for each service touchpoint were computed based on core evaluations. Using Equation (14), the failure risk value for each failure mode was calculated. This value represents the ranking of the service touchpoints for the smart office desk, driven by smart technology, as shown in Table 9.

**Figure 5:**
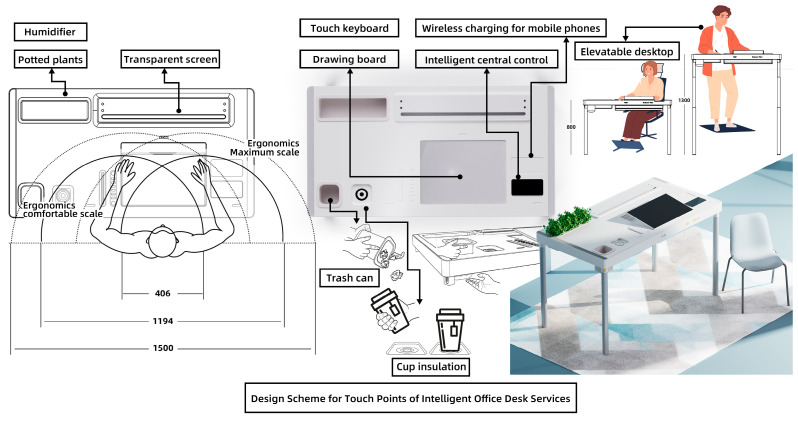
Service touchpoint design plan for office desk driven by smart technology.

Based on the office desk service touchpoint design plan displayed in Table 5 and the failure risk analysis results shown in Table 9, the design plan was finalized. The design plan is illustrated in Figure 5.

### 6.3. Case Analysis

Based on the design plan proposed in this paper and created using the BEDFITA model, the positions of each functional module were rearranged according to user behavior habits. Less frequently used items, such as the desktop trash bin and the cup insulation module, are placed in the lower-left corner. The desktop green plant is combined with the humidifier module, with the green plant helping to alleviate the user’s emotions and the humidifier providing water for the plant.

Using ergonomic user parameters, the maximum and optimal interaction ranges for the user are planned. The screen, drawing pad, keyboard, wireless phone charging module, and smart central control interface are positioned in front of the user, on the right half of the desk. This arrangement allows the user to view and control all the smart desk’s functional modules through the smart central control interface. Moreover, the design plan is compared to five existing office desk design solutions, and the comparison results are displayed in Table 10.

Based on the comparison, the office desk service touchpoint design plan derived from the BEDFITA model offers a superior user experience. First, its thoughtful arrangement of functional modules helps users stay more organized in their office activities. Second, by addressing emotional needs, the design creates a more relaxed and happier work environment. Finally, the incorporation of smart central control technology allows users to operate the desk more effectively.

Therefore, the smart desk service touchpoint design plan developed using the BEDFITA model stands out from most existing design plans, offering an enhanced user experience. This model offers valuable guidance for designing product service touchpoints powered by smart technology, improving user experience, reliability, and feasibility in such designs.

## 7. Conclusions

This paper highlights the complexity and diversity inherent in designing product service touchpoints within the context of smart technology. Traditional design procedures and methods often fall short in meeting user needs and enhancing user experience due to this complexity and diversity. In response, this paper introduces the BEDFITA model, designed to create product service touchpoint design plans that better align with user needs.

The BEDFITA model establishes a comprehensive service touchpoint design logic, centered on user needs and beginning with specific user behaviors. By analyzing users’ complete behavior during product use or activity performance, the model identifies the emotions associated with each behavior node. Using the Kano model’s logic, user needs are matched, and product functions are developed based on these needs, resulting in a function plan that closely aligns with user requirements. This plan is then used to map out user experience behaviors, followed by service touchpoint design and a failure risk analysis.

The BEDFITA model’s design logic is closely connected to user behavior, both during the product function planning stage and the service touchpoint design stage. Consequently, service touchpoint designs generated using the BEDFITA model tend to outperform those produced by traditional design methods, effectively addressing the complexity and diversity introduced by smart technology. This model offers designers an effective approach and framework for service touchpoint design.

However, as smart technology evolves, users’ cognitive load is likely to increase. Future research should consider the integration of user knowledge and smart technology. Additionally, due to the differences in user groups, usage scenarios, and product functions, the design of product service touchpoints driven by smart technology will also vary. These aspects represent potential areas for further refinement of the BEDFITA model.

## Figures and Tables

**Table 1:** User demand matching table.

User Emotions		Without This Behavior				
		Satisfied	Normal	Indifferent	Tolerable	Dissatisfied
**With this behavior**	**Satisfied**	Ss	Hp	Hp	Hp	Lk
	**Normal**	Ng	Id	Id	Id	Nr
	**Indifferent**	Ng	Id	Id	Id	Nr
	**Tolerable**	Ng	Id	Id	Id	Nr
	**Dissatisfied**	Ng	Ng	Ng	Ng	Ss

**Table 2:** Fuzzy Evaluation Term Set.

Evaluation Terms	Severity (S)	Occurrence (O)	Detection (D)	Fuzzy Number
Very high	Almost No Impact	Almost Never Occurs	Directly Determinable	(L, N, U)
High	Main Functions Operate, Some Minor Functions Decrease	Occurs Rarely	Determined After Simple Testing	(L, N, U)
Middle	Main Functions Decrease, Some Minor Functions Malfunction	Occasionally Occurs	Determined After Professional Testing	(L, N, U)
Low	Main Functions Malfunction, Most Minor Functions Malfunction	Frequently Occurs	Difficult to Determine with Professional Testing	(L, N, U)
Very low	System Stops Operating	Very Likely to Occur	Indeterminable	(L, N, U)

**Table 3:** User Office Behavior Analysis Results.

**Behavior Node**	**B** **_1_**	**B** **_2_**	**B** **_3_**	**B** **_4_**	**B** **_5_**
User Behavior	Place Personal Items	Power on desktop electronic office devices	Pour Water into Cup (Multiple Times)	Work (Seated Posture)	Dispose of Desktop Trash
**Behavior Node**	**B** **_6_**	**B** **_7_**	**B** **_8_**	**B** **_9_**	**B** **_10_**
User Behavior	Take a Short Break Away from Desk (Brief)	Work (standing posture)	Relieve Stress	Check Schedule	Adjust Humidifier
**Behavior Node**	**B** **_11_**	**B** **_12_**	**B** **_13_**	**B** **_14_**	**B** **_15_**
User Behavior	Adjust Lighting	Adjust sitting posture (multiple times)	Organize Documents	Clean Desktop	Adjust Audio
**Behavior Node**	**B** **_16_**	**B** **_17_**	**B** **_18_**	**B** **_19_**	
User Behavior	Have Snacks and Drinks	Interact with colleagues	Organize the Workstation	Shut Down Desktop Electronic Office Devices	

**Table 4:** Office Desk Function Construction Plan.

**User Demand Ranking**	**B_3_ (Ud_3_ = 1.24)**	**B_6_ (Ud_6_ = 0.98)**	**B_10_ (Ud_10_ = 0.86)**	**B_4_ (Ud_4_ = 0.74)**	**B_8_ (Ud_8_ = 0.72)**
Corresponding Demand	Needs to control the temperature of the water cup	Wants to flexibly adjust the desk height	Hopes to control the air humidity around the workstation	Wants to be more focused during work	Wishes to relieve work pressure
Function	(F1) Cup heating or cooling function	(F2) Automatic desktop height adjustment function	(F3) Humidification function	(F4) Intelligent central control function	(F5) Stress relief function
**User Demand Ranking**	**B** **_18_** **(Ud** **_18_ = 0.66)**	**B** **_9_** **(Ud** **_9_ = 0.58)**	**B** **_13_** **(Ud** **_13_ = 0.49)**	**B** **_17_** **(Ud** **_17_ = 0.44)**	**B** **_12_** **(Ud** **_12_ = 0.3)**
Corresponding Demand	Wants workstation organization to be simple	Wants itinerary recording and reminders	Wishes to minimize the accumulation of paper documents	Desires convenient communication with colleagues	Wants to easily adjust sitting posture
Function	(F_6_) Modularity of functions	(F_7_) Quick recording of itineraries and temporary work information	—	—	Automatic desktop height adjustment function
**User Demand Ranking**	**B** **_14_** **(Ud** **_14_ = 0.29)**	**B** **_5_** **(Ud** **_5_ = 0.19)**	**B** **_7_** **(Ud** **_7_ = 0.18)**	**B** **_19_** **(Ud** **_19_ = 0.17)**	**B_1_ (Ud** **_1_ = 0.16)**
Corresponding Demand	Wants the desktop to be easy to clean	Desires easy disposal of desktop trash	Wants to work standing for long periods	Needs optimization of electronic device power buttons	—
Function	—	(F_8_) Desktop small trash bin	Desktop automatic height adjustment function	—	—
**User Demand Ranking**	**B_2_ (Ud_2_ = 0.13)**	**B_11_ (Ud_11_ = 0.1)**	**B_16_ (Ud_16_ = 0.09)**	**B_15_ (Ud_15_ = 0)**	
Corresponding Demand	—	—	—	—	
Function	—	—	—	—	

**Table 5:** Preliminary Service Touchpoint Design Plan for Office Desk.

**Number**	**Id_1_**	**Id_2_**	**Id_3_**	**Id_4_**	**Id_5_**
User Interaction Behaviors	Place Files/Items	Turn On Electronic Devices	Place Water Cup	Set High-Efficiency Work Mode	Adjust Desktop Height
Service Touchpoint Design	(P_1_) Specific Placement Location	(P_2_) Smart Central Control Screen	(P_3_) Cup Warming Module	Smart Central Control Screen	Smart Central Control Screen
**Number**	**Id_6_**	**Id_7_**	**Id_8_**	**Id_9_**	**Id_10_**
User Interaction Behaviors	Work (Seated Posture)	Turn on Humidifier	Relax Mood	Dispose of Desktop Trash	Work (Standing Posture)
Service Touchpoint Design	(Mouse + Keyboard)	Smart Central Control Screen	(P_4_) Small Green Plants	(P_5_) Small Desk-side Trash Bin	(P_6_) Height-Adjustable Desk Legs
**Number**	**Id_11_**	**Id_12_**	**Id_13_**	**Id_14_**	**Id_15_**
User Interaction Behaviors	Work (Seated Posture)	Plan Temporary Work	Online Meeting	Take a Break Away from Desk	Work (Seated Posture)
Service Touchpoint Design	Smart Central Control Screen	Smart Central Control Screen	(P_7_) Device Ports	—	(Mouse + Keyboard)
**Number**	**Id_16_**	**Id_17_**	**Id_18_**	**Id_19_**	**Id** **_20_**
User Interaction Behaviors	Print Documents	Text Work	Plan Next Day’s Work	Turn Off Electronic Devices	Leave Workstation
Service Touchpoint Design	—	(P8) Writing Instruments	Smart Central Control Screen	Smart Central Control Screen	—

**Table 6:** Core User Weights for the Office Desk.

Core User Group	Career	Severity (S)	Occurrence (O)	Detection (D)
Core user group 1	Free Creator	0.35	0.22	0.36
Core user group 2	Engineer	0.16	0.34	0.15
Core user group 3	Designer	0.41	0.27	0.18
Core user group 4	College Teacher	0.008	0.17	0.31

**Table 7:** Failure Mode Analysis of Office Desk Service Touchpoints.

Service Touchpoints	Failure Mode	Failure Reason	Failure Consequence
(P_1_) Specific Placement Location	Not convenient for placing files/items	Unreasonable desktop modularization settings	Wrong or forgotten files/items
(P_2_) Smart Central Control Screen	Intelligent central control screen error control	Accidentally touching the intelligent central control screen while working	Causing work errors
(P_3_) Cup Warming Module	The water temperature is too high or too low	Temperature setting error of water cup insulation module	Personnel burns or inappropriate water temperature
(P_4_) Small Green Plants	Not convenient to manage	Green plants selected for soil cultivation	Low desktop cleanliness
(P_5_) Small Desk-side Trash Bin	It is not easy to throw out garbage	Unreasonable desktop modularization settings	User-generated troubles
(P_6_) Height-Adjustable Desk Legs	Table leg adjustment is not smooth	Table legs are stuck	The office process is uncomfortable
(P_7_) Device Ports	Equipment connection is inconvenient	Unreasonable position of device socket	Restrict user device usage
(P_8_) Writing Instruments	Paper waste is prone to accumulation	There is a high demand for paper-based office work	Drafts and official documents are easily confused

**Table 8:** Fuzzy Numbers for Failure Risk of Office Desk Service Touchpoints.

Service Touchpoints	Sw	Ow	Dw
(P_1_) Specific Placement Location	(6.54, 8.08, 9.13)	(5.41, 6.99, 8.57)	(5.51, 7.08, 8.65)
(P_2_) Smart Central Control Screen	(5.18, 6.68, 8.06)	(4.25, 6.01, 7.75)	(2.09, 3.66, 5.23)
(P_3_) Cup Warming Module	(4.53, 6.24, 7.95)	(5.67, 7.33, 8.51)	(2.33, 4.05, 5.76)
(P_4_) Small Green Plants	(5.41, 6.99, 8.57)	(6.85, 8.35, 9.34)	(1.98, 3.09, 4.55)
(P_5_) Small Desk-side Trash Bin	(7.25, 8.75, 9.53)	(4.85, 6.49, 8.16)	(3.84, 5.34, 6.84)
(P_6_) Height-Adjustable Desk Legs	(6.17, 7.82, 8.79)	(4.82, 6.51, 8.17)	(1.37, 2.97, 4.57)
(P_7_) Device Ports	(4.56, 6.27, 7.97)	(4.91, 6.47, 8.23)	(1.25, 2.46, 4.03)
(P_8_) Writing Instruments	(4.25, 6.08, 7.74)	(6.65, 8.24, 9.07)	(1.56, 3.21, 4.86)

**Table 9:** Failure Risk Analysis Results for the Office Desk Service Touchpoint Design Plan.

Touchpoint Number	QSw	DOw	QDw	Rw
P_1_	7.41	6.46	6.56	314.02
P_5_	8.00	5.96	4.84	230.77
P_4_	6.46	7.68	2.84	140.90
P_3_	5.67	6.62	3.48	130.62
P_8_	5.42	7.46	2.67	107.96
P_2_	6.14	5.42	3.14	104.49
P_6_	7.05	5.96	2.44	102.52
P_7_	5.70	5.96	2.18	74.06

**Table 10:** Comparison Between the Service Touchpoint Design Plan and Existing Products.

Existing Plan	Service Touchpoints	Comparison Results
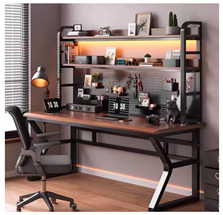	Trapezoidal table legs, desktop storage rack, desk lamp	This design does not have height-adjustable functionality, meaning that the user’s working posture is fixed, which may cause discomfort over time and affect health. The desktop storage shelf occupies a large portion of the desk surface, limiting the user’s range of movement. Additionally, the desk lamp is used infrequently.
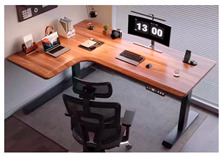	Adjustable table legs, L-shaped tabletop, control board	The L-shaped desktop divides the desk into two areas: a working area and a document storage area. However, this design only meets the user’s basic office needs, and the user needs to provide additional office supplies to meet other requirements.
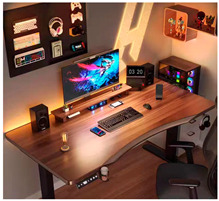	Adjustable table legs, control board, cup holder, computer peripheral storage rack	The control panel is located on the left side of the desk, making it inconvenient for the user to control. The cup holder restricts the size of the cup the user can use. The computer peripheral storage rack is mounted on the nearby wall, which limits its usability to some extent.
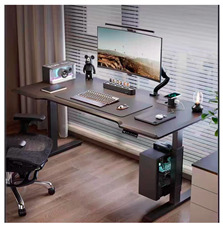	Adjustable table legs, computer case mounting bracket, control board	This design features a relatively reasonable modular setup and includes a fixed module for the computer case. However, it still only meets the user’s basic office needs, and the user needs to provide additional office supplies to meet other requirements.
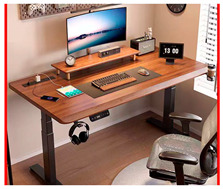	Adjustable table legs, inserts, control board, cup holder, headphone hook	The control panel is still located on the left side, reducing the convenience of user operation. Although a power strip structure was added to the desk, providing convenience for using electronic devices, the excessive number of chargers for electronic products makes the desktop cluttered and difficult to manage.

## Data Availability

https://pan.baidu.com/s/108qwQYbKHyYED6PtxhCsMA=1920 (accessed on 23 April 2024).
